# Study on the microbial diversity of ear canal secretions from patients with otomycosis

**DOI:** 10.3389/fsurg.2024.1277799

**Published:** 2024-02-09

**Authors:** Zhuxiang Chen, Zhang Zhao

**Affiliations:** Department of Otorhinolaryngology, Hubei NO.3 People's Hospital of Jianghan University, Wuhan, China

**Keywords:** otomycosis, fungi, bacteria, microbial community, diversity

## Abstract

Otomycosis is caused by fungi, which usually cause discharge and additional discomfort. The highest incidence of otomycosis occurs in summer. To better treat this disease, it is necessary to study the microbial diversity of otomycosis secretions. In this regard, this study used high-throughput sequencing technology to determine the microbial diversity of the ear canal secretions of six typical patients with otomycosis in Wuhan via internal transcribed spacer (ITS) and 16S rRNA analyses and proposed a reasonable clinical treatment plan. Six patients with otomycosis in the Department of Otorhinolaryngology, Hubei Third People's Hospital Affiliated with Jianghan University, were selected from June 2022 to August 2022. The results showed that *Staphylococcus* spp. (average abundance 29.05%) was the dominant bacteria and *Aspergillus* spp. (average abundance 90.68%) was the dominant fungus involved in otomycosis secretion. *Aspergillus* spp. can cause inflammation of the external auditory canal combined with bacterial infections such as *Staphylococcus* spp., which can cause discharge in the ear canal. High-throughput sequencing provides comprehensive information on the microbial community involved in otomycosis discharge and will aid in evaluating the efficacy of clinical treatment and medication.

## 1 Introduction

Otomycosis, also known as otitis externa mycotica, is caused by the invasion or massive reproduction of opportunistic fungi and the production of secretions, increasing the complexity of treatment (1). Otomycosis is a common inflammatory lesion in otolaryngology that accounts for 10%–20% of ear canal infections (2). Otomycosis is very common in tropical and subtropical regions where the temperature and humidity are high (3). Frequent ear picking (4), chronic otitis media (5), and the use of antibiotics (6) all increase the incidence of otomycosis. Otomycosis can also cause symptoms such as ear itching, hearing impairment, and ear pain (7).

*A. niger* or *A. flavus* complex in the genus *Aspergillus* spp., and *Candida albicans*, *Candida parapsilosis* in the genus *Candida* spp. have been reported to be common causative agents of otomycosis (8, 9). However, the fungal community involved in otomycosis varies with region, climate and patient immune status (10). For example, *Aspergillus tubeingensis* is the dominant and most common isolated species in Western China and Southern Ireland (11, 12), and *A. tubingensis* is most common in southern Iran (13). *C.albicans* is more common in immunocompromised patients with otomycosis than in immunocompromised patients without otomycosis (14). Fungal infection lesions can sometimes completely cover the eardrum, causing hearing loss that is difficult to heal on its own (15). Fungal infection is more persistent and easier to relapse, so effective treatment measures should be taken in a timely manner. When treating otomycosis, the first step is to diagnose the condition, which usually involves examining the ear canal to look for signs of fungal infection, such as redness, swelling, discharge, or specific types of fungus. After diagnosis, the first step is to clear the ear canal containing the fungus and secretions. Second, the selection of appropriate antifungal drugs is crucial for the treatment of otomycosis, usually based on the type of fungus (such as *Candida* spp. or *Aspergillus* spp.) and drug resistance. Clotrimazole (16), miconazole (17) and tolnaftate (18) are currently commonly used drugs for the treatment of otomycosis. In some cases, topical antifungal ear drops may be needed.

Clinically, determining what kind of opportunistic fungal infection is involved and whether bacteria are involved are highly important for the early diagnosis and treatment of otomycosis. At present, opportunistic fungi are mainly identified clinically by isolation, culture, and microscopic examination based on fungal morphology (19). However, the above methods have several limitations, such as equipment conditions, personnel level, and staining methods (20). Moreover, the culture process easily results in contamination by pathogenic bacteria, and traditional methods can identify only the main pathogenic bacteria. Therefore, it is difficult to accurately identify pathogenic bacteria based on the abovementioned traditional methods. In recent years, with the rise of high-throughput sequencing technology, culture-free methods have been gradually introduced for the identification of pathogenic microorganisms (21). To date, high-throughput sequencing has not been widely used in the study of otomycosis. In this study, high-throughput sequencing technology was used to study the fungal and bacterial communities involved in otomycosis to provide a reference for clinical treatment.

## 2 Materials and methods

### 2.1 Ethics and consent

Patients with otomycosis were recruited from June 2022 to August 2022 from the Department of Otorhinolaryngology, Hubei Third People's Hospital Affiliated with Jianghan University. This study was approved by the Ethics Committee of the Third People's Hospital of Hubei Province Affiliated with Jianghan University (No. 2023010), and written informed consent was obtained from each patient participating in this study.

### 2.2 Sample collection

In this study, 6 patients with otomycosis who met the inclusion criteria (all had a history of ear picking, no otitis media, and no history of diabetes) were recruited. Otomycosis was diagnosed through ear canal observation, direct smears, and culture samples, and all 6 patients had ear canal discharge. The ear secretions from one ear were collected, and the patients were not treated with other drugs before collection (See Table 1 for details). Ear canal secretions were collected using sterile cotton swabs soaked in sterile saline, packed into sterile sampling tubes, and stored in a −80°C freezer, after which DNA extraction and polymerase chain reaction (PCR) amplification were performed.

**Table 1:** Detailed clinical information of 6 patients.

Number	Gender	Age	Symptom	Duration
Sample 1	Male	35	Ear fullness, itching	1 week
Sample 2	Male	38	Ear fullness, itching	2 weeks
Sample 3	Male	41	Earache, ear fullness, itching	3 weeks
Sample 4	Female	42	Itchy ears, tinnitus	2 weeks
Sample 5	Female	28	Itchy ears, tinnitus	1 week
Sample 6	Female	41	Itchy ears, tinnitus	2 weeks

### 2.3 DNA extraction, PCR amplification, library construction and sequencing

Fungal DNA and bacterial DNA were extracted from the samples using a FastDNA® Spin Kit for Soil (American Mpbio Corporation) (22) following the manufacturer's protocol to ensure that the DNA extracted from each sample met the requirements. DNA quality was checked using 2% agarose gel electrophoresis, and the concentration and purity of the DNA were detected using a NanoDrop 2000 spectrophotometer (Thermo Fisher Scientific, Wilmington, DE, USA). Next, the ITS1F-ITS2R region of the fungal internal transcriptional spacer gene was amplified using primers (ITS 1F 5′-CTTGGTCATTTAGAGGAAGTAA-3′; ITS 2R 5′-GCTGCGTTCTTCATCGATGC-3′) (23); the V3-V4 region of the bacterial 16S rRNA gene was amplified using primers (338F 5′-ACTCCTACGGGAGGCAGCAG-3′; and 806R 5′-GGACTACHVGGGTWTCTAAT-3′) (24). Amplified products were purified using an AxyPrep DNA Gel Extraction Kit (Axygen Biosciences, Union City, CA, USA) for library construction. The library was subsequently sequenced on the Illumina NextSeq 2000 platform of Shanghai Meiji Biomedical Technology Co., Ltd. The original data were uploaded to the NGDC database with the upload number CRA011427.

### 2.4 Data analysis

The bacterial diversity was clustered using the USEARCH11-uparse algorithm, the OTU sequence similarity was 0.97, and the species classification database was silva138/16s_bacteria, with a classification confidence of 0.7. The fungal diversity was clustered using the USEARCH11-uparse algorithm, the operational taxonomic unit (OTU) sequence similarity was 0.97, and the species classification database was unite8.0/its_fungi, with a classification confidence of 0.7. R software (version 4.1.1) was used to calculate the diversity of bacteria and fungi, and BugBase was used to predict the phenotype of bacteria in the samples (https://bugbase.cs.umn.edu/index.html).

## 3 Results and discussion

### 3.1 Sequencing results

**Figure 1:**
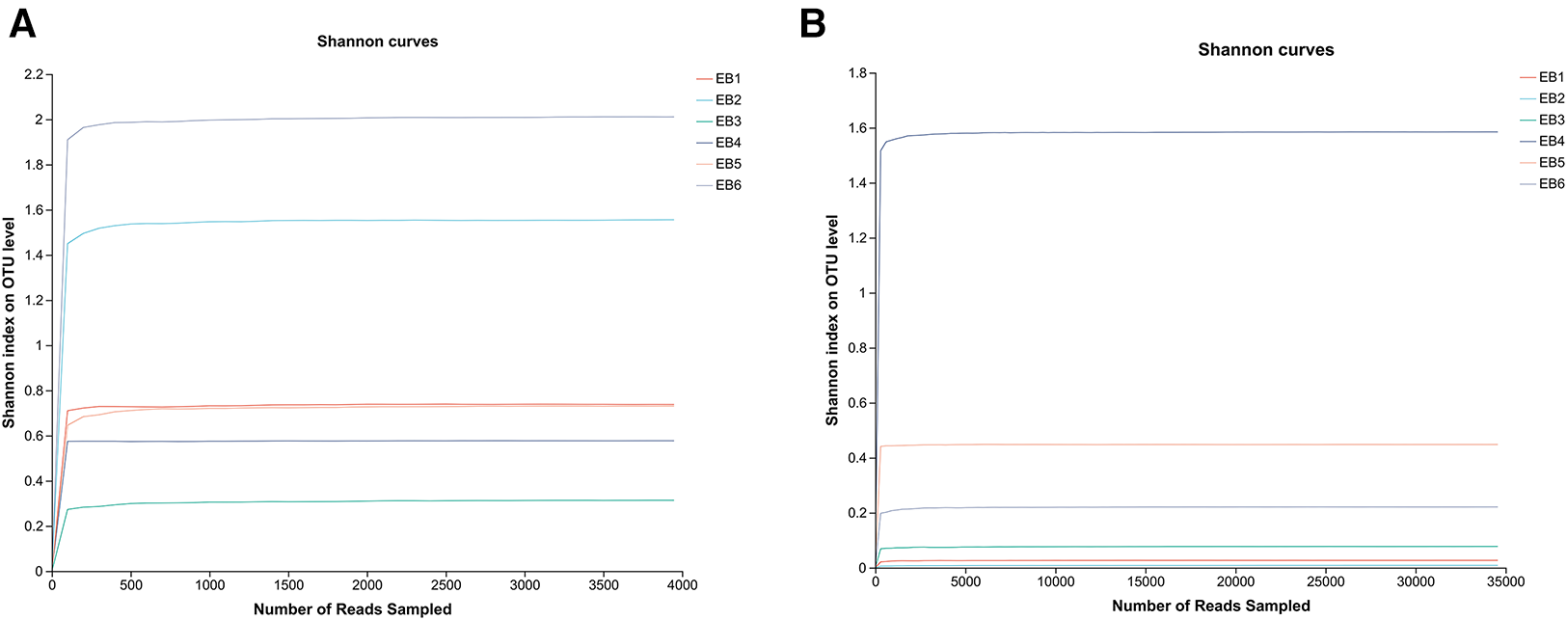
Shannon dilution curves (**A**) for bacteria, (**B**) for fungi.

OTUs are used to classify and compare microorganisms, while the Shannon index is an ecological index that measures community diversity. In microbial community analysis, Shannon dilution curves are often used to evaluate the richness and evenness of species diversity in a sample. The OTU Shannon dilution curves of both fungi and bacteria in the otomycosis secretion samples tended to flatten (as shown in Figures 1A,B), indicating that the detection rate of microbial communities in the otomycosis secretion samples was close to saturation, and that the amount of the current sequencing could cover most of the species in the sample. This means that the sequencing performed was sufficient to cover most species in the sample, providing a reliable basis for subsequent analysis (25).

### 3.2 Bacterial diversity

**Figure 2:**
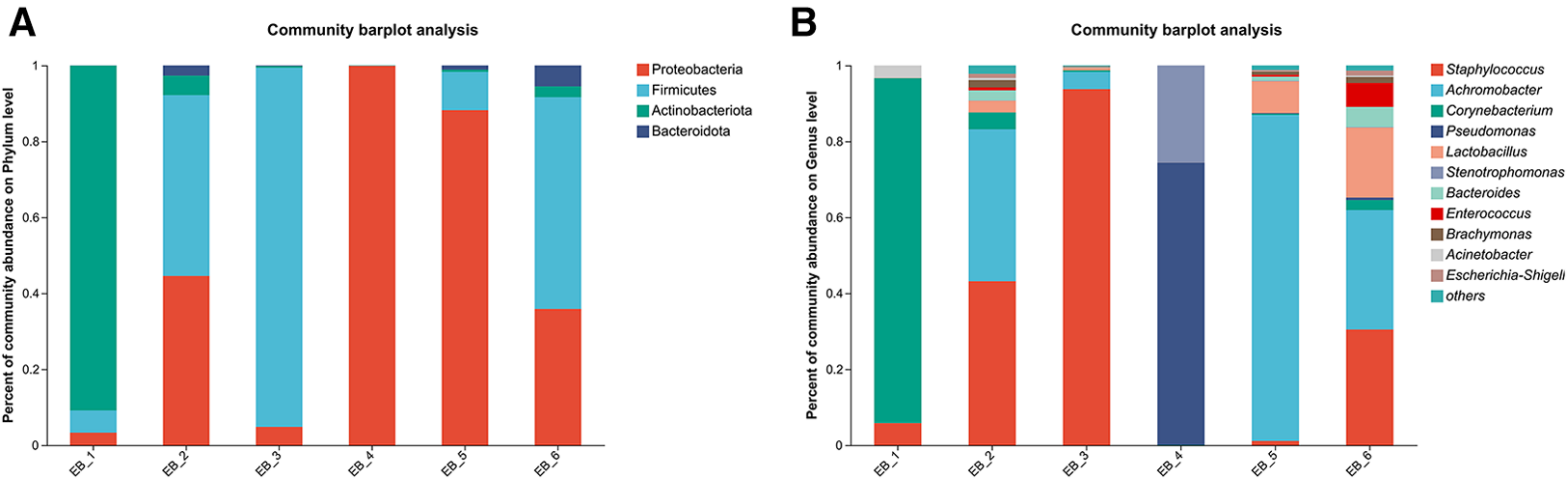
Phylum- and genus-level community structure map of otomycosis secretions. Different colors represent different phyla and genera, and the colored blocks on the right are sorted in descending order of abundance. (**A**) The community structure of the main bacteria at the phylum level. Proteobacteria, Firmicutes, and Actinobacteria were the dominant phyla. (**B**) The community structure of the main bacteria at the genus level. *Staphylococcus* spp., *Achromobacter* spp., *Corynebacterium* spp., *Pseudomonas* spp., and *Lactobacillus* spp. were the dominant genera.

After quality control filtering and removal of chimeric sequences, a total of 257,429 sequences were generated, and 228,551 sequences were obtained after optimization, with an average length of 425 bp. These findings demonstrated that the sequencing depth was sufficient to provide reliable bacterial community analysis and was deep enough to reveal the microbial diversity in the samples (26). Approximately ≥97% of the sequences were clustered into one OTU, and the species OTUs with sequence numbers ≥5 in at least 3 samples were retained. The species OTUs with a total sequence ≥20 were retained, and the OTUs aligned to the mitochondrial sequence were removed and flattened according to the minimum sample sequence. A total of 29 OTUs were obtained. The dominant bacterial phyla (abundance >1%) were Proteobacteria, Firmicutes, and Actinobacteria, and the dominant genera were *Staphylococcus* spp., *Achromobacter* spp., *Corynebacterium* spp., *Pseudomonas* spp., and *Lactobacillus* spp. (as shown in Figure 2A). Among them, *Staphylococcus* spp. is a common pathogen and a pus-forming bacterium that was detected in 5 samples, and the average abundance was 29.05% (as shown in Figure 2B). The high abundances of Proteobacteria, Firmicutes, Actinobacteria, etc., indicate that these bacteria play a dominant role in the ear canal microbial community. Identification of dominant genera such as *Staphylococcus* spp., *Achromobacter* spp., *Corynebacterium* spp., *Pseudomonas* spp., and *Lactobacillus* spp. can aid in understanding the pathological mechanisms of ear canal infection (27). *Staphylococcus aureus*, *Staphylococcus epidermidis*, *Pseudomonas aeruginosa*, coagulase-negative *Staphylococcus* spp. and *Klebsiella pneumoniae* are common bacteria in the ear canal secretions of patients with suppurative otitis media (28); *S. epidermidis* is a normal bacterium in the ear canal (29); and the rest are pathogenic bacteria, which is similar to the results of this study. The high abundance of *Staphylococcus* spp. and other bacteria in ear canal secretions indicates their important role in ear canal infections. Identifying these bacteria can help develop targeted treatment plans (30). Additionally, possible bacterial infection should be considered in patients with otomycosis who clinically do not respond well to conventional antifungal drugs. The coexistence of bacteria and fungi may complicate treatment, so identification and treatment of these pathogenic bacteria should be considered during treatment (31).

**Figure 3:**
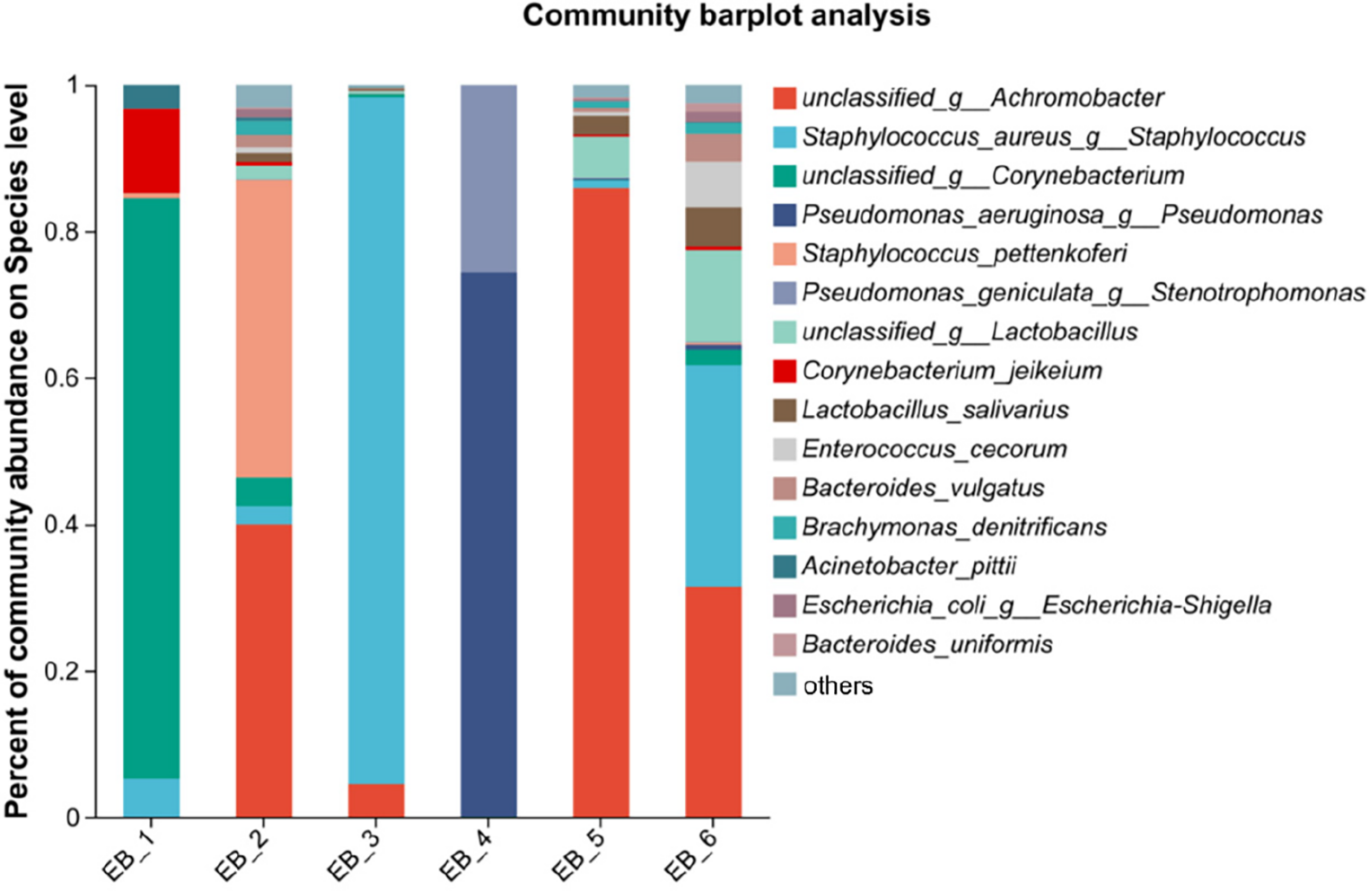
Species-level community structure diagram of otomycosis secretions.

**Figure 4:**
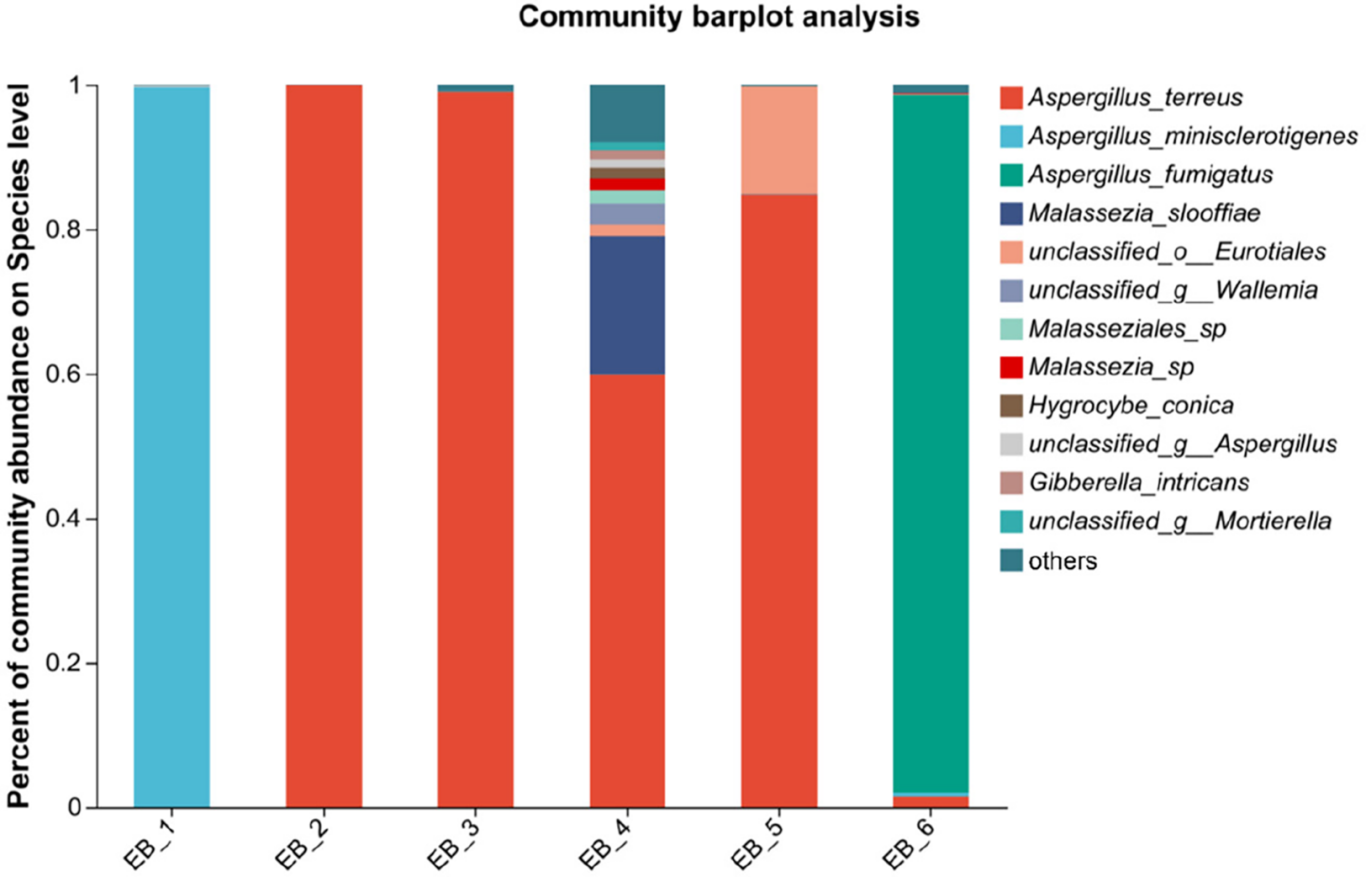
Species-level community structure diagram of otomycosis secretions.

At the species level (as shown in Figure 3), *S. aureus* was present in 3 out of the 6 samples, ranking second in average abundance (the average abundance was 22.12%). Chen et al. (5) showed that methicillin-resistant *S. aureus* is the main pathogen of otomycosis. Methicillin-resistant *S. aureus* is a major cause of bacterial infections (bacteremia, endocarditis, soft tissue infections, hospital-acquired infections, etc.) in hospital and community settings (32). *P. aeruginosa* was found in only sample 4. According to the analysis shown in Figure 4, *P. aeruginosa* significantly increased the diversity of fungi, increasing the complexity of clinical treatment. The next step should be to isolate and characterize the most abundant unidentified bacterial species. In addition, the interaction between bacteria and fungi is worthy of further exploration.

### 3.3 Fungal diversity

**Figure 5:**
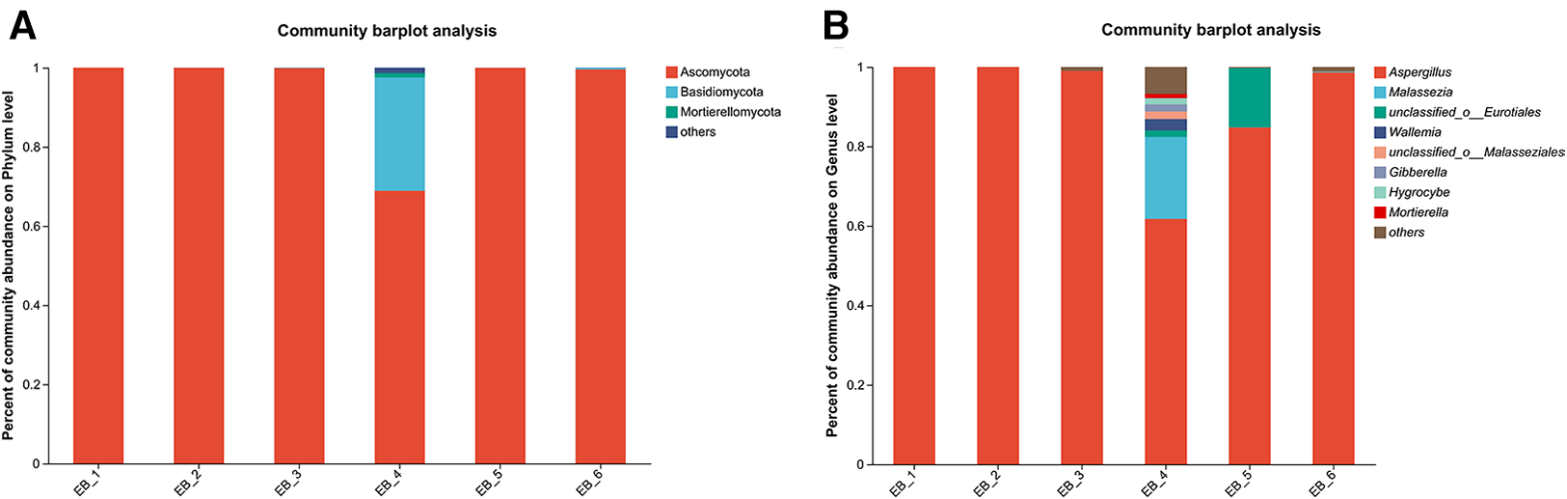
Phylum- and genus-level community structure diagram of otomycosis secretions. Different colors represent different phyla and genera, and the colored blocks on the right are sorted in descending order of abundance. (**A**) The community structure of the main fungi at the phylum level; the dominant phylum was Ascomycota. (**B**) The community structure of the main fungi at the genus level; the dominant genus was *Aspergillus* spp.

After quality control filtering and removal of chimeric sequences, a total of 252,964 sequences were generated, and 239,866 sequences were optimized, with an average length of 261 bp. ≥97% of the sequences were clustered into one OTU and smoothed according to the minimum sample sequence; a total of 80 OTUs were obtained. The dominant phylum was Ascomycota, and the dominant genus was *Aspergillus* spp.(average abundance 90.68%) (as shown in Figures 5A,B); these findings are consistent with the results of Gu et al. (7) on patients with otomycosis in Nanjing, Jiangsu Province, China. *Aspergillus* spp. is the dominant genus involved in otomycosis in southern China. This study highlights the importance of geographic location in determining human microbial community structure; the dominance of *Aspergillus* spp. in otomycosis in southern China may be related to environmental factors and ecosystem characteristics. According to recent studies, otomycoses are commonly caused by the *A. niger* complex and yeasts (*Candida*). Notably, the *A. niger* or *A. flavus* complex are the major fungal species causing otomycosis. The above studies highlight the complexity of otomycosis and the challenges in their treatment (33).

At the species level, four of the six samples were *Aspergillus terreus* (average abundance 57.48%), one was *Aspergillus minisclerotigenes* (average abundance 16.73%), and one was *Aspergillus fumigatus* (average abundance 16.10%) (as shown in Figure 4). The results of Zhang et al. (12) showed that the most common fungal inflammation in the external auditory canal was caused by *A. terreus* (50.9%), whereas *A. fumigatus* was less common (9.1%), similar to the results of this study. *A. terreus* occurs in a variety of environments and is the fourth leading cause of invasive and noninvasive aspergillosis (34). *A. fumigatus* is a saprophytic fungus found primarily in soil that can also cause invasive airborne infections. In immunocompromised patients, these infections are often fatal (35, 36). *A. fumigatus* occurs less frequently in otomycoses but remains an important pathogen. There are no reports about otomycosis caused by *A. minisclerotigenes*. Studies have shown that *A. minisclerotigenes* can cause fungal keratitis (37). This suggest that, although currently understudied, the impact of *A. minisclerotigenes* should also be paid attention to during the treatment of otomycosis. Therefore, if otomycosis is clinically diagnosed, the scabs and secretions in the external auditory canal of the patient should be thoroughly removed, and antifungal medication should be applied to allow the infection to act as an antifungal agent, thereby eliminating the symptoms of otomycosis. When treating otomycosis, it is important to consider that different *Aspergillus* spp. may have different susceptibilities to antifungal drugs. For different pathogenic bacteria, different therapeutic strategies should be adopted, and not all antifungal drugs can be used for the treatment of otomycosis; for example, among *Aspergillus* spp., only *A. terreus* is intrinsically resistant to amphotericin B (32). Next, the pathogenesis of otomycosis caused by *A. minisclerotigenes* and the effect of existing drugs on its treatment can be investigated.

### 3.4 Bacterial phenotype prediction

The results of the bacterial phenotype prediction showed that most of the bacteria were aerobic (aerobic bacteria), and the proportion of anaerobic bacteria was relatively low, which was in line with the actual environment of the ear canal. The microbial community of the ear canal is typically dominated by aerobic bacteria, consistent with the situation in which the ear canal is an aerated and drier environment. Aerobic bacteria grow and reproduce more easily in such an environment. The proportion of anaerobic bacteria in the ear canal was lower. This may be due to the natural physiological conditions of the ear canal that limit the growth of anaerobic bacteria (27). In addition, otomycosis secretions have a strong biofilm-forming ability (38), which is consistent with the dominance of *Staphylococcus* spp. in terms of bacterial diversity. A biofilm is a protective layer formed by microorganisms, such as bacteria and fungi, that helps them survive in harsh environments. This biofilm formation is particularly important in ear canal infections because it can lead to persistent and difficult-to-treat ear canal infections (39). *Achromobacter* spp. contributed 40.15% to the phenotype of bacterial biofilm formation. *Corynebacterium* spp. and *Pseudomonas* spp. contributed 15.97% and 9.32%, respectively, to the observed bacterial biofilm formation. The contribution of *Acinetobacter* spp. to biofilm formation was significant, consistent with its role in hospital-acquired infections. *Achromobacter* spp. are multidrug-resistant bacteria that can survive in hospital settings and cause serious infections, such as hospital-acquired pneumonia. *Corynebacteria* spp. and *Pseudomonas* spp. also play important roles in the formation of biofilms. The biofilm-forming ability of these bacteria is a key factor in their adaptation and survival in ear canal infections (40). In addition, most bacteria have a certain potential pathogenicity (mainly *Achromobacter* spp., 40.15%), which should receive increased attention in clinical treatment; that is, the harm caused by pathogenic bacteria cannot be ignored when treating fungal infections. Studies have shown that *Achromobacter* spp. can form biofilms (41, 42) and cause diseases, such as hospital-acquired pneumonia (HAP) (43), which is consistent with the predictive results of this phenotype. The potential pathogenicity of *Achromobacter* spp. needs to be taken seriously in clinical treatment. When treating otomycosis, doctors need to be aware of the harm caused by these bacteria. Because these bacteria may be resistant to multiple antibiotics, choosing the appropriate antibiotic and treatment regimen is critical for controlling and preventing the spread of infection (44).

## 4 Conclusion

In this study, high-throughput sequencing was used to identify the microbial community of the ear canal secretions of 6 patients with otomycosis of the external auditory canal in Wuhan, Hubei Province. The sequencing results showed that the dominant genus of fungi was *Aspergillus* spp., and the dominant genus of bacteria was *Staphylococcus* spp. The results provide comprehensive information on the microbial community of patients with otomycosis of the external auditory canal, which can provide a reference for clinical treatment.

## Data Availability

The datasets presented in this study can be found in online repositories. The names of the repository/repositories and accession number(s) can be found in the article/Supplementary Material.

## References

[1] Mao C Li A Hu J Wang P Peng D Wang J Efficient and accurate diagnosis of otomycosis using an ensemble deep-learning model. Front Mol Biosci. (2022) 9:951432. 10.3389/fmolb.2022.951432 36060244 PMC9437247

[2] Kiakojuri K Mahdavi Omran S Roodgari S Taghizadeh Armaki M Hedayati MT Shokohi T Molecular identification and antifungal susceptibility of yeasts and molds isolated from patients with otomycosis. Mycopathologia. (2021) 186:245–57. 10.1007/s11046-021-00537-1 33718990

[3] Alarid-Coronel J Celis-Aguilar E Escobar-Aispuro L Muñoz-Estrada V. Otomycosis in immunocompetent patients: clinical and mycological features. Our experience with 40 cases. Clin Otolaryngol. (2017) 43(1):373–7. 10.1111/coa.12966 28834405

[4] Huang W Li Y Huang J Luo Y Huang N. Endoscope ear pick: an emerging but neglected medical device. Front Med (Lausanne). (2022) 9:977554. 10.3389/fmed.2022.977554 36457570 PMC9705332

[5] Chen C-H Wang C-Y Cheng M-Y Hsih W-H Tien N Chou C-H Definite therapy of mixed infection alleviates refractory dilemma of adult chronic suppurative otitis media. J Microbiol Immunol Infect. (2022) 55:1283–92. 10.1016/j.jmii.2022.07.014 36117089

[6] Alshahni MM Alshahni RZ Fujisaki R Tamura T Shimizu Y Yamanishi C A case of topical ofloxacin-induced otomycosis and literature review. Mycopathologia. (2021) 186:871–6. 10.1007/s11046-021-00581-x 34410567

[7] Gu X Cheng X Zhang J She W. Identification of the fungal community in otomycosis by internal transcribed spacer sequencing. Front Microbiol. (2022) 13:820423. 10.3389/fmicb.2022.820423 35369424 PMC8965282

[8] Tasić-Otašević S Golubović M Đenić S Ignjatović A Stalević M Momčilović S Species distribution patterns and epidemiological characteristics of otomycosis in Southeastern Serbia. J Mycol Med. (2020) 30:101011. 10.1016/j.mycmed.2020.101011 32693980

[9] Sukumar B Premamalini T Shree SN Kindo AJ. P022 luliconazole—a novel potent imidazole activity against *Aspergillus* niger and *Aspergillus* flavus causing otomycosis. Med Mycol. (2022) 60:22. 10.1093/mmy/myac072.p022

[10] Westby D O'Connell N Powell J Fenton JE. The changing nature of paediatric otomycosis in the Mid-West of Ireland. J Laryngol Otol. (2020) 134:592–6. 10.1017/s0022215120001164 32713390

[11] Zhang LL Wang X Houbraken J Mei H Deng S. Molecular identification and in vitro antifungal susceptibility of *Aspergillus* isolates recovered from otomycosis patients in Western China. Mycopathologia. (2020) 185:527–35. 10.1007/s11046-020-00448-7 32346838

[12] Zhang S Jin M Hu S Zhang Y Zhou G. Administration of 1% topical voriconazole drops was effective and safe in the treatment of refractory otomycosis without tympanic membrane perforation. Ann Otol Rhinol Laryngol. (2020) 130:273–9. 10.1177/0003489420946783 32772544

[13] Javidnia J Ghotbi Z Ghojoghi A Solhjoo K Alshahni MM Jeddi SA Otomycosis in the south of Iran with a high prevalence of tympanic membrane perforation: a hospital-based study. Mycopathologia. (2022) 187:225–33. 10.1007/s11046-022-00626-9 35347533

[14] Pooja G Kirtivardhan V O'Horo JC Aditya S. Fungal culture diagnostic stewardship: an avenue for antimicrobial stewardship in the immunocompromised host. Open Forum Infect Dis. (2019) 6:148. 10.1093/ofid/ofz360.342

[15] Gheorghe DC Niculescu A-G Bîrcă AC Grumezescu AM. Nanoparticles for the treatment of inner ear infections. Nanomaterials. (2021) 11:1311. 10.3390/nano11051311 34067544 PMC8156593

[16] Kiakojuri K Rajabnia R Mahdavi Omran S Pournajaf A Karami M Taghizadeh Armaki M. Role of clotrimazole in prevention of recurrent otomycosis. BioMed Res Int. (2019) 2019:5269535. 10.1155/2019/5269535 31950041 PMC6944967

[17] Nemati S Gerami H Faghih Habibi A Kazemnejad E Shabani Asl N Aghsaghloo V Sertaconazole versus clotrimazole and miconazole creams in the treatment of otomycosis: a placebo-controlled clinical trial. Iran J Otorhinolaryngol. (2022) 34:27–34. 10.22038/ijorl.2021.54805.2872 35145933 PMC8801007

[18] Jimenez-Garcia L Celis-Aguilar E Díaz-Pavón G Muñoz Estrada V Castro-Urquizo Á Hernández-Castillo N Efficacy of topical clotrimazole vs. topical tolnaftate in the treatment of otomycosis. A randomized controlled clinical trial. Braz J Otorhinolaryngol. (2020) 86:300–7. 10.1016/j.bjorl.2018.12.007 30826311 PMC9422661

[19] de Souza Costa P Prado A Bagon NP Negri M Svidzinski TIE. Mixed fungal biofilms: from mycobiota to devices, a new challenge on clinical practice. Microorganisms. (2022) 10:1721. 10.3390/microorganisms10091721 36144323 PMC9506030

[20] Lu G Wang Z Zhang B Zhou Z Hu D Zhang D. Detecting forest musk deer abscess disease pathogens using 16S rRNA high-throughput sequencing technology 13:3142. Animals (Basel). (2023) 13(19):3142. 10.3390/ani13193142 37835748 PMC10572063

[21] Miller S Chiu C. The role of metagenomics and next-generation sequencing in infectious disease diagnosis. Clin Chem. (2021) 68:115–24. 10.1093/clinchem/hvab173 34969106

[22] Bou Orm E Sauvagère S Rocher J Benezet J-C Bayle S Siatka C Estimating the bias related to DNA recovery from hemp stems for retting microbial community investigation. Appl Microbiol Biotechnol. (2023) 107:4665–81. 10.1007/s00253-023-12582-5 37227475

[23] Adams RI Miletto M Taylor WJ Bruns TD. Dispersal in microbes: fungi in indoor air are dominated by outdoor air and show dispersal limitation at short distances. ISME J. (2013) 7:1262–73. 10.1038/ismej.2013.28 23426013 PMC3695294

[24] Xu N Tan G Wang H Gai X. Effect of biochar additions to soil on nitrogen leaching, microbial biomass and bacterial community structure. Eur J Soil Biol. (2016) 74:1–8. 10.1016/j.ejsobi.2016.02.004

[25] Hughes JB Hellmann JJ Ricketts TH Bohannan BJ. Counting the uncountable: statistical approaches to estimating microbial diversity. Appl Environ Microbiol. (2001) 67:4399–406. 10.1128/aem.67.10.4399-4406.2001 11571135 PMC93182

[26] Caporaso JG Lauber CL Walters WA Berg-Lyons D Huntley J Fierer N Ultra-high-throughput microbial community analysis on the illumina HiSeq and MiSeq platforms. ISME J. (2012) 6:1621–4. 10.1038/ismej.2012.8 22402401 PMC3400413

[27] Brook I. The role of bacterial interference in otitis, sinusitis and tonsillitis. Otolaryngol Head Neck Surg. (2005) 133:139–46. 10.1016/j.otohns.2005.03.012 16025067

[28] Kaźmierczak W Janiak-Kiszka J Budzyńska A Nowaczewska M Kaźmierczak H Gospodarek-Komkowska E. Analysis of pathogens and antimicrobial treatment in different groups of patients with chronic otitis media. J Laryngol Otol. (2022) 136:219–22. 10.1017/s0022215121003224 34702380

[29] Méric G Mageiros L Pensar J Laabei M Yahara K Pascoe B Disease-associated genotypes of the commensal skin bacterium *Staphylococcus* epidermidis. Nat Commun. (2018) 9:5034. 10.1038/s41467-018-07368-7 30487573 PMC6261936

[30] Post JC. Direct evidence of bacterial biofilms in otitis media. Laryngoscope. (2015) 125(9):2003–14. 10.1002/lary.25291 26297170

[31] Marom T Nokso-Koivisto J Chonmaitree T. Viral-bacterial interactions in acute otitis media. Curr Allergy Asthma Rep. (2012) 12:551–8. 10.1007/s11882-012-0303-2 22968233 PMC3493715

[32] Turner NA Sharma-Kuinkel BK Maskarinec SA Eichenberger EM Shah PP Carugati M Methicillin-resistant *Staphylococcus* aureus: an overview of basic and clinical research. Nat Rev Microbiol. (2019) 17:203–18. 10.1038/s41579-018-0147-4 30737488 PMC6939889

[33] Bojanović M Ignjatović A Stalević M Arsić-Arsenijević V Ranđelović M Gerginić V Clinical presentations, cluster analysis and laboratory-based investigation of *Aspergillus* otomycosis—a single center experience. Journal of Fungi. (2022) 8:315. 10.3390/jof8030315 35330316 PMC8948793

[34] Vahedi Shahandashti R Lass-Flörl C. Antifungal resistance in *Aspergillus* terreus: a current scenario. Fungal Genet Biol. (2019) 131:103247. 10.1016/j.fgb.2019.103247 31247322

[35] Latgé J-P Chamilos G. *Aspergillus* fumigatus and aspergillosis in 2019. Clin Microbiol Rev. (2019) 33. 10.1128/cmr.00140-18 PMC6860006 31722890

[36] Arastehfar A Carvalho A Houbraken J Lombardi L Garcia-Rubio R Jenks JD *Aspergillus* fumigatus and aspergillosis: from basics to clinics. Stud Mycol. (2021) 100:100115. 10.1016/j.simyco.2021.100115 34035866 PMC8131930

[37] Karimizadeh EM Eslampoor A Dolatabadi S Najafzadeh MJ Houbraken J. First case of fungal keratitis due to *Aspergillus* minisclerotigenes in Iran. Current Medical Mycology. (2019) 5:45. 10.18502/cmm.5.2.1162 31321339 PMC6626709

[38] Ballah FM Islam MS Rana ML Ferdous FB Ahmed R Pramanik PK Phenotypic and genotypic detection of biofilm-forming *Staphylococcus* aureus from different food sources in Bangladesh. Biology (Basel). (2022) 11:949. 10.3390/biology11070949 36101330 PMC9311614

[39] Costerton JW Stewart PS Greenberg EP. Bacterial biofilms: a common cause of persistent infections. Science. (1999) 284:1318–22. 10.1126/science.284.5418.1318 10334980

[40] Percival SL Suleman L Vuotto C Donelli G. Healthcare-associated infections, medical devices and biofilms: risk, tolerance and control. J Med Microbiol. (2015) 64:4. 10.1099/jmm.0.000032 25670813

[41] Cameron LC Bonis B Phan CQ Kent LA Lee AK Hunter RC. A putative enoyl-CoA hydratase contributes to biofilm formation and the antibiotic tolerance of *Achromobacter* xylosoxidans. NPJ Biofilms Microbiomes. (2019) 5:20. 10.1016/10.1038/s41522-019-0093-6 31396394 PMC6684605

[42] Sandri A Saitta GM Veschetti L Boschi F Passarelli Mantovani R Carelli M In vivo inflammation caused by *Achromobacter* spp. cystic fibrosis clinical isolates exhibiting different pathogenic characteristics. Int J Mol Sci. (2023) 24:7432. 10.3390/ijms24087432 37108596 PMC10139000

[43] Chao L Fei P Jun G Weifeng Y Yi J Changting L Hospital acquired pneumonia due to *Achromobacter* spp. in a geriatric ward in China: clinical characteristic, genome variability, biofilm production, antibiotic resistance and integron in isolated strains. Front Microbiol. (2016) 7:621. 10.3389/fmicb.2016.00621 27242678 PMC4860489

[44] Peleg AY Seifert H Paterson DL. *Acinetobacter* baumannii: emergence of a successful pathogen. Clin Microbiol Rev. (2008) 21:3. 10.1128/cmr.00058-07 PMC2493088 18625687

